# Ratio-tuned green-synthesized Ag–Fe bimetallic nanoparticles embedded in electrospun gelatin/chitosan nanofibrous scaffolds for antibacterial applications

**DOI:** 10.1039/d6ra01562j

**Published:** 2026-05-14

**Authors:** Nour Alhuda Asad, Fatih Erci, Fatma Bayram

**Affiliations:** a Department of Chemical Engineering, Konya Technical University Konya Turkey fsariipek@ktun.edu.tr +90 552 936 5062; b Department of Biotechnology, Necmettin Erbakan University Konya Turkey; c Science and Technology Research and Application Center (BITAM), Necmettin Erbakan University Konya Turkey

## Abstract

Wound and device-associated infections caused by drug-resistant microorganisms are a major global health problem, increasing morbidity, mortality and economic burden. Antimicrobial nanofibrous scaffolds have therefore gained attention as wound dressings capable of reducing infection risk. In this study, gelatin/chitosan (GEL/CTS) nanofibrous scaffolds incorporating silver–iron bimetallic nanoparticles (Ag–FeNPs) synthesized *via* a green route using *Hypericum perforatum* (*H. perforatum*) leaf extract were developed as potential antibacterial wound-dressing materials. Ag–FeNPs were produced at room temperature and characterized by different analytical techniques. Microstructural analysis confirmed that the biogenic nanoparticles were spherical, with an average size of 60.98 ± 18.09 nm, and were uniformly distributed throughout the GEL/CTS fibers. Ag–FeNPs with varying Ag : Fe ratios were subsequently embedded into the polymeric matrix to enhance scaffold wettability and antibacterial performance. FT-IR, XRD, FE-SEM, and water-contact-angle measurements demonstrated that incorporation of CTS and Ag–FeNPs reduced the fiber diameter (171.47 ± 55.96 nm) and improved hydrophilicity, with the GEL/CTS/Ag–Fe (2 : 1) formulation exhibiting the lowest WCA value (60.72°). Antibacterial assays conducted against *Staphylococcus aureus* (*S. aureus*) and *Escherichia coli* (*E. coli*) revealed that the green-synthesized Ag–FeNPs were active against both strains, with stronger inhibition observed for *E. coli*. Among the nanofibrous scaffolds, the sample containing the highest Ag composition yielded the most pronounced reduction in bacterial colony formation. Overall, these findings indicate that GEL/CTS scaffolds reinforced with Ag–FeNPs possess favorable structural and biological properties, which highlighting their potential as effective antibacterial materials for applications such as wound dressings.

## Abstract 2

Green-synthesized Ag–Fe bimetallic nanoparticles embedded in electrospun gelatin/chitosan nanofibrous scaffolds exhibited enhanced antibacterial activity and promising potential for wound-dressing applications.

## Graphical abstract

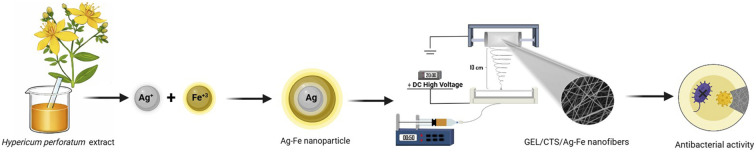

## 1. Introduction

Nanomaterials, which are characterized by dimensions ranging from 1 to 100 nm, have attained a prominent position across diverse applications due to their enhanced surface-to-volume ratio. Nanomaterials have become one of the most interesting and important research areas in modern nanotechnology due to their distinct physical, chemical, and catalytic characteristics.^1,2^ Metallic nanoparticles have gained significant prominence among nanomaterials across various application fields. Green synthesis, which uses biomaterials, especially plant extracts, has recently gained popularity as an alternative to the traditional physical and chemical methods used for the synthesis of metallic nanoparticles. By minimizing the use of toxic agents compared to chemical synthesis, biological approaches offer more environmentally benign alternative.^3^ Among the nanoparticles, silver nanoparticles (AgNPs) have emerged as one of the most thoroughly researched nanomaterials, largely due to their potent biological functionalities such as antimicrobial, antioxidant, and cytotoxic effects.^4–6^ Notably, AgNPs synthesized *via* green routes stand out due to their enhanced biocompatibility, making them particularly advantageous for biological applications.^7^ On the other hand, iron nanoparticles (FeNPs) are distinguished by their biocompatibility, low toxicity, high catalytic efficiency, and superparamagnetic properties.^8^ Emerging research has increasingly focused on bimetallic nanoparticles (BMNPs) as advanced alternatives to their monometallic counterparts. While BMNPs are fabricated *via* conventional top-down methodologies, they offer superior functionalities arising from synergistic effects. This synergy enables the fine-tuning of physicochemical properties, including increased surface reactivity, robust stability, and optimized biocompatibility, which collectively broaden their application potential.^9,10^

In recent years, nanofibers have garnered substantial attention due to their extraordinary properties compared to conventional fibrous structures. As a prominent class of nanomaterials, nanofibers exhibit superior characteristics, such as a high surface area-to-volume ratio, low density, unique porosity reaching up to 90%, interconnected pore architectures, and the feasibility of chemical functionalization. These unique physicochemical characteristics offer great promise for a number of biomedical applications, especially in tissue engineering, drug delivery, and biosensors.^11,12^ Although various techniques exist for nanofiber fabrication, electrospinning is considered the most versatile, straightforward, and cost-effective method.^13^ In a standard electrospinning setup, a liquid jet is ejected from a Taylor cone *via* electrical forces, subsequently stretched, and solidified into fibers. The efficiency of nanofiber production is significantly influenced by material properties, such as conductivity and concentration, as well as processing parameters, including voltage, flow rate, and tip-to-collector distance.^14^

Bio-based polymeric materials have recently been the focus of extensive research because of their environmental compatibility, renewability, and inherent biodegradability.^15^ In this regard, chitin has garnered considerable attention as the most prevalent natural polymer. Predominantly sourced from crustaceans, insects, and fungi, it undergoes chemical or enzymatic deacetylation to yield chitosan, a derivative widely utilized in the biomedical field.^16^ Chitosan-based bionanocomposites are extensively utilized in tissue engineering, drug delivery, and wound/burn dressings due to their biocompatibility, non-toxicity, and versatility. Furthermore, chitosan stands out in the food industry as an antimicrobial packaging agent and emulsifier; these functionalities are attributed to the presence of both hydrophilic and hydrophobic moieties within its molecular structure.^17^ In addition to chitosan (CTS), gelatin (GEL) is widely utilized in biomedical applications. As an FDA-approved natural polymer, gelatin exhibits high biocompatibility, biodegradability, and hydrophilicity, while demonstrating low immunogenicity and minimal toxicity. Produced through the hydrolysis of collagen, GEL can be easily modified due to the functional groups on its surface, and its structure supports both cell proliferation and fluid diffusion.^17,18^ The integration of antimicrobial chitosan with cell-adhesive gelatin provides an exceptional composite platform for tissue engineering scaffolds. This is particularly relevant as the efficacy of materials in biomedical contexts is largely determined by their ability to biomimic the native extracellular matrix (ECM).^19,20^

In the present study, bimetallic Ag–FeNPs were synthesized *via* a green chemistry approach using *H. perforatum* extract as both a reducing and stabilizing agent. *H. perforatum* is a medicinal plant well known for its antibacterial, antioxidant, and anti-inflammatory activities, which are highly relevant to wound healing and infection control applications.^21,22^*H. perforatum* offers the advantage of established biomedical relevance alongside multifunctional biological activity, providing a rational basis for its selection in this study. The synthesized NPs using *H. perforatum* extract with different Ag : Fe molar ratios were subsequently incorporated into GEL/CTS nanofiber scaffolds fabricated through electrospinning. Although the antibacterial activity of individual Ag or FeNPs has been previously reported, the effect of incorporating bimetallic Ag–FeNPs with varying Ag : Fe ratios into GEL/CTS nanofibers on antibacterial performance remains largely unexplored. The integration of Ag–FeNPs was specifically aimed at enhancing the scaffolds' surface wettability and synergistic antibacterial performance. To evaluate the impact of the bimetallic composition on the final composite, both the nanoparticles and the NP-loaded nanofibrous mats underwent characterization. The structural, morphological, and physicochemical properties were systematically analyzed using different analytical techniques. Furthermore, the antibacterial efficacy of the individual NPs and the resulting composite scaffolds were evaluated against *Staphylococcus aureus* and *Escherichia coli*, providing new insights for the rational design of biopolymeric nanofibrous scaffolds for antibacterial applications.

## 2. Experimental section

### 2.1. Materials

The biopolymeric matrix for this work consisted of type B gelatin (from bovine skin approx. 75 bloom, Sigma-Aldrich, USA), and medium molecular weight CTS (*M*_w_ = 300 000 g mol^−1^ and degree of deacetylations >75%, Sigma-Aldrich). Silver nitrate (AgNO_3_, Sigma-Aldrich) and iron nitrate (Fe(NO_3_)_3_, Sigma-Aldrich), which are salts of silver and iron, were used for synthesis of antibacterial bimetallic nanomaterial. Acetic acid (AC, Merck) and ethanol (Eth, Sigma-Aldrich) were employed as solvents. Citric acid (CA, Merck) was used as a crosslinker. All chemicals were of analytical grade and utilized as received.

### 2.2. Preparation of plant extract

Freshly air-dried leaves of *H. perforatum* were thoroughly rinsed several times with double-distilled water to remove adhering dust and surface impurities. The cleaned leaves were then dried at ambient temperature until all residual moisture had completely evaporated. The dried leaves were finely ground into a homogeneous powder. A quantity of 6 g of this powder was immersed in 240 mL of hot distilled water and maintained under continuous magnetic stirring at 80 °C for 2 h for effective phytoconstituent extraction. The resultant suspension was allowed to stand overnight to facilitate complete extraction and then filtered through Whatman No. 1 filter paper. The obtained filtrate, representing the aqueous leaf extract of *H. perforatum*, was stored in the dark at 4 °C.

### 2.3. Biosynthesis of silver–iron bimetallic nanoparticles

Initially, Ag–Fe precursor solutions were formulated in the specific molar ratios listed in Table 1 using 1 M AgNO_3_ and 1 M Fe(NO_3_)_3_ stock solutions. The resulting mixture was magnetically stirred for 60 min to ensure homogeneous blending. Subsequently, 60 mL of freshly prepared *H. perforatum* extract was gradually introduced into the Ag–Fe mixture under continuous agitation and maintained at a constant stirring condition for approximately 2 h to facilitate nucleation and growth of nanoparticles.

**Table 1:** Composition of the synthesized Ag–Fe NP samples

Sample	Content of AgNO_3_ (mM)	Content of Fe(NO_3_)_3_ (mM)
AgNPs	10	0
Ag–FeNPs (2 : 1)	10	5
Ag–FeNPs (1 : 1)	10	10
Ag–FeNPs (1 : 2)	5	10
FeNPs	0	10

**Fig. 1:**
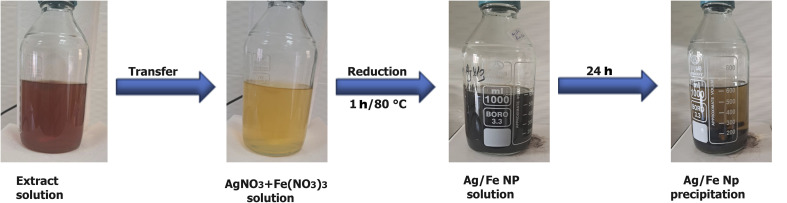
Formation of a dark brownish solution after mixing AgNO_3_ and Fe(NO_3_)_3_ with *H. perforatum* leaf extract under magnetic stirring at 80 °C for 1 h, and its appearance after 24 h.

The reaction progress was monitored *via* a distinct color transition from pale brown to deep brown, signifying the bioreduction of Ag^+^ and Fe^3+^ ions to their metallic states (as seen in Fig. 1). The formation of Ag–Fe NPs was confirmed *via* UV-vis spectroscopy. Following the synthesis, the resulting colloidal dispersion was subjected to an aging process in darkness for 24 h for nanoparticle stabilization. The synthesized suspension was then filtered through Whatman Grade 5 (2.5 µm) filter paper to remove residues. The resulting filtrate was centrifuged at 10 000 rpm for 20 min, followed by a rigorous purification sequence. The precipitate was washed once with ultrapure deionized water and subsequently thrice with ethanol to facilitate the removal of unreacted ions and residual organic impurities. Subsequently, the recovered nanoparticles were oven-dried at 80 °C and preserved in amber glass vials at 4 °C to maintain stability for subsequent characterization and application phases. A schematic illustration of the biogenic synthesis route of Ag–Fe NPs is presented in Fig. 1.

### 2.4. Fabrication of biopolymeric nanofibrous scaffolds

Biopolymeric nanofibrous scaffolds composed of GEL, GEL/CTS, and GEL/CTS/Ag–Fe NPs were fabricated following the preparation of their respective electrospinning solutions. Initially, a 15% (w/v) pure GEL solution was prepared in a 50% (v/v) acetic acid (AC) solvent system and stirred continuously at 45 °C for 1 h, followed by 24 h of magnetic stirring at room temperature to obtain a homogeneous solution.

For the preparation of GEL/CTS nanofibers, separate GEL and CTS solutions were first formulated. The CTS solution (0.01 g mL^−1^) was prepared by dissolving chitosan in a 50% (v/v) acetic acid aqueous solution under vigorous magnetic stirring at 45 °C for 4 h. The GEL solution (0.1 g mL^−1^) was formulated under identical experimental conditions. Subsequently, the two solutions were blended in a 1 : 1 (v/v) ratio and homogenized *via* continuous stirring for 48 h to ensure the formation of a uniform GEL/CTS precursor mixture.

To synthesize GEL/CTS/Ag–FeNPs nanofibrous scaffolds, the preformed GEL/CTS solution was incorporated with Ag–FeNPs possessing different Ag and Fe compositions at a concentration of 5 wt% relative to the total polymer weight. The Ag–FeNPs suspensions were first dispersed in an AC–water binary solvent system and ultrasonicated for 60 min to achieve uniform dispersion and prevent agglomeration. These nanoparticle suspensions were subsequently added to the GEL/CTS matrix and magnetically stirred for 48 h to ensure complete homogenization.

The resulting electrospinning solutions were transferred into 5 mL syringes equipped with blunt 21G stainless steel needles and mounted on a syringe pump. Electrospinning was performed using an Inovenso top-down electrospinning system under optimized process parameters: an applied voltage of 20 kV, a feed rate of 0.5 mL h^−1^, and a tip-to-collector distance of 10 cm. The compositions of the fabricated biopolymeric nanofibers are summarized in Table 2.

**Table 2:** WCA measurements and average fiber diameters of GEL, GEL/CTS, and GEL/CTS/Ag–Fe nanofibers

Sample	Mass ratios of GEL/CTS (wt%)	Content of NP relative to the total polymer (wt%)	WCA values (°)	Average fiber diameters (nm)
GEL	15/0	0	45.82	206.97 ± 77.67
GEL/CTS	10/1	0	66.15	102.86 ± 55.36
GEL/CTS/Ag	10/1	5	49.27	150.34 ± 62.94
GEL/CTS/Ag–Fe (2 : 1)	10/1	5	60.72	171.47 ± 55.96
GEL/CTS/Ag–Fe (1 : 1)	10/1	5	75.57	179.34 ± 52.34
GEL/CTS/Ag–Fe (1 : 2)	10/1	5	89.94	185.34 ± 59.07
GEL/CTS/Fe	10/1	5	108.43	184.34 ± 57.14

**Fig. 2:**
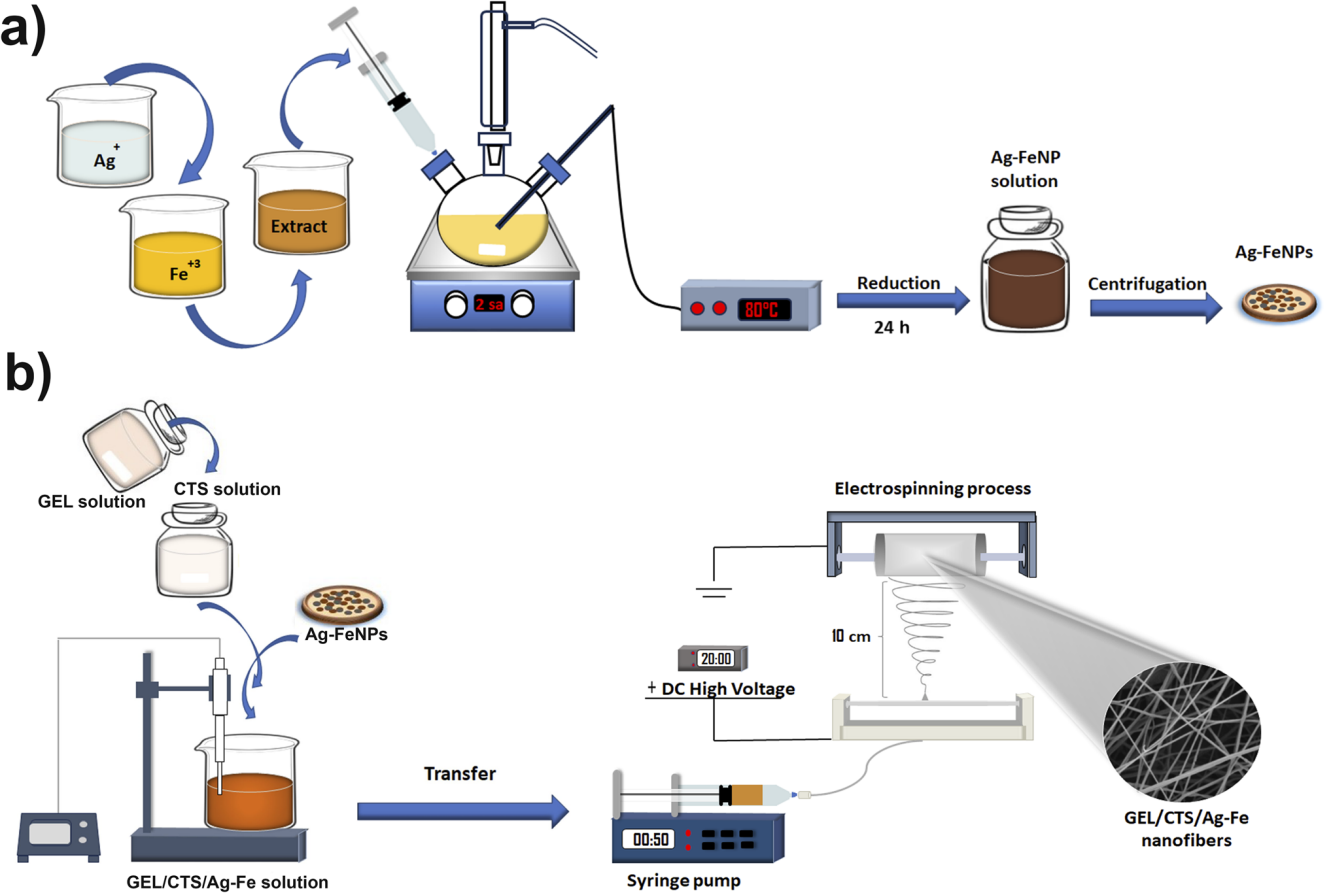
A schematic illustration of the fabrication process of (a) Ag–FeNPs and (b) GEL/CTS/Ag–Fe nanofibrous scaffolds.

Following electrospinning, the obtained nanofibrous mats were dried overnight in a vacuum oven at 37 °C to remove residual solvent. A schematic illustration of the fabrication process of GEL/CTS/Ag–Fe nanofibers is presented in Fig. 2.

### 2.5. Characterization of nanoparticle and nanofibrous scaffold samples

The morphological features and chemical composition of the synthesized nanoparticles and electrospun nanofibrous scaffolds were characterized using a combination of advanced analytical techniques. Fourier-transform infrared (FT-IR) spectra were obtained with a Shimadzu IR Prestige-21 spectrometer, while optical absorption analyses were performed using a Shimadzu UV-1700 UV-vis spectrophotometer. The surface morphology and structural integrity of the nanofibers were examined through field-emission scanning electron microscopy (FE-SEM; Zeiss SEM 500).

The crystalline phases of the nanoparticles was analyzed using X-ray diffraction (XRD; PANalytical Empyrean) equipped with Cu Kα radiation (*λ* = 1.54 Å, 40 kV) across a diffraction angle (2*θ*) range of 5°–80°. The average crystallite size of Ag domains was estimated from the Ag(111) reflection using the Scherrer equation.

The detailed morphology and particle size of the Ag–FeNPs were further explored *via* transmission electron microscopy (TEM; JEOL JEM-2100). Particle size distributions of the Ag–FeNPs and fiber diameter measurements of GEL/CTS/Ag–Fe scaffolds were quantitatively analyzed using ImageJ software.

To evaluate the wettability behavior of the electrospun scaffolds, static water contact angle (WCA) measurements were conducted at ambient temperature using a contact angle goniometer (Biolin Scientific Attension, Theta Lite). For each sample, 4 µL of distilled water was gently placed on the nanofiber surface, and six replicate measurements were recorded to determine the mean WCA value.

### 2.6. Antibacterial activity tests of Ag–FeNPs and scaffold samples

The antibacterial efficacy of the synthesized nanoparticles was evaluated using the agar well diffusion method. Gram-positive *Staphylococcus aureus* (ATCC 25923) and Gram-negative *Escherichia coli* (ATCC 25922) were utilized as model microorganisms. Initially, lyophilized cultures were inoculated into Nutrient Broth (NB) containing 20% glycerol to prepare stock cultures, which were stored at −18 °C to maintain viability. For the experiments, colonies from the stock cultures were transferred to fresh NB and incubated overnight at 35 °C. Bacterial suspensions were prepared and adjusted to a 0.5 McFarland turbidity standard. Following the adjustment, these cultures were uniformly swabbed onto Mueller–Hinton Agar (MHA) plates. Then, wells were precisely excised into the agar medium. Subsequently, 100 µL of the nanoparticle suspension was introduced into each well after being sonicated for 30 min to ensure homogeneous dispersion. The plates were then incubated at 37 °C for 16 h to allow for bacterial growth. Following the incubation period, the diameters of the inhibition zones around the wells were measured in millimeters to determine the antibacterial activity. All procedures were performed in triplicate.

The antibacterial activity of the biosynthesized AgNPs was evaluated *via* the broth microdilution method. To determine the minimum inhibitory concentration (MIC), AgNPs were prepared in a concentration range of 0.78–200 µg mL^−1^ within 96-well microplates containing Mueller–Hinton Broth (MHB). Each well was inoculated with the target bacteria at a density of 10^5^–10^6^ CFU mL^−1^. Following a 24 h incubation at 35 °C, bacterial growth was quantified by measuring absorbance at 600 nm using a microplate reader.

The antibacterial performance of the nanofibrous mats against *S. aureus* and *E. coli* was evaluated by monitoring changes in optical density (O.D.) within a liquid medium. Each nanofibrous mat weighing 20 mg was exposed to 5 mL of a NB bacterial suspension with an initial O.D. of 0.17 at 600 nm. The samples were then incubated in a shaking incubator at 37 °C for 6 h to facilitate interaction between the fibers and the bacteria. After the incubation period, the O.D. of the bacterial suspensions was measured at 600 nm using a spectrophotometer to quantify growth inhibition. All measurements were conducted in triplicate, and the results were recorded as the mean values.

### 2.7. Statistical analysis

Statistical analysis of the quantitative data was performed using one-way analysis of variance (ANOVA). All values are presented as mean ± standard deviation, and statistical significance was accepted at *p* < 0.05.

## 3. Results and discussion

### 3.1. Characterization of green-synthesized Ag–FeNPs

**Fig. 3:**
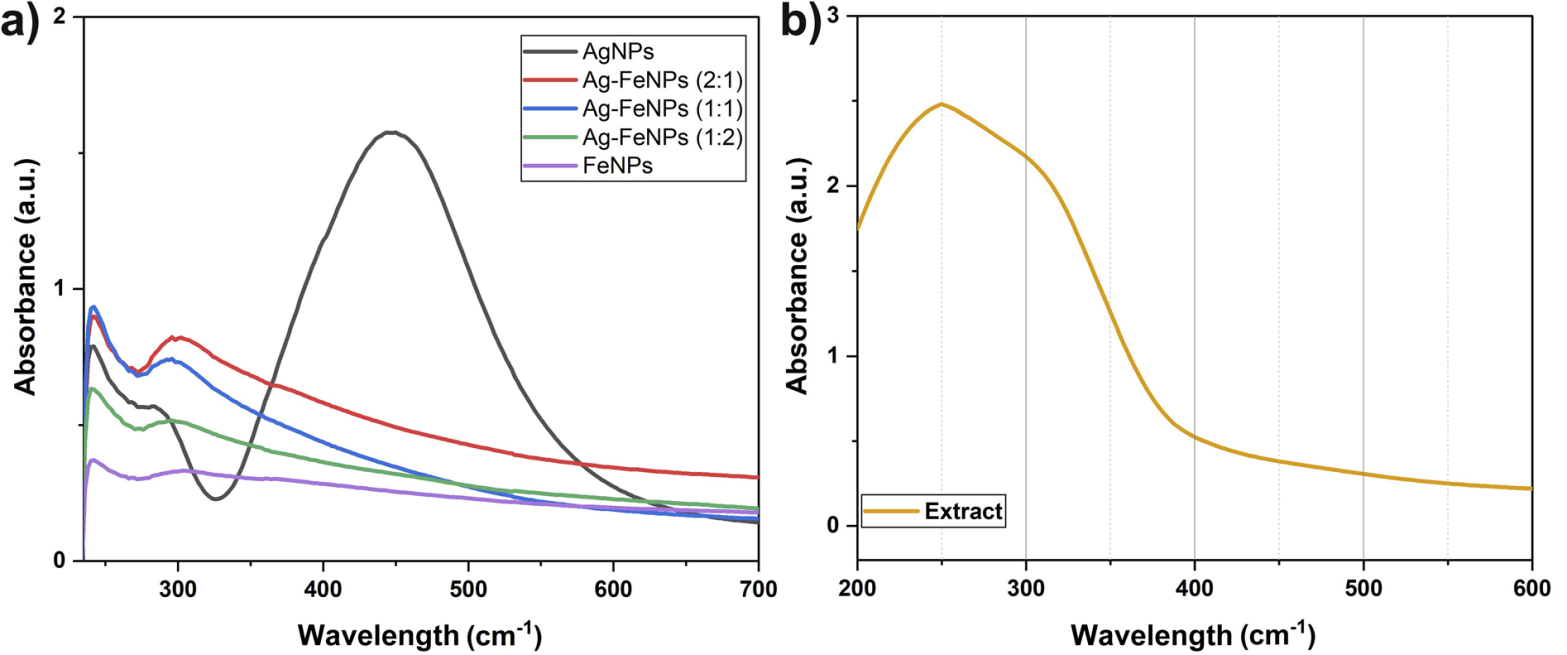
UV-vis spectra of (a) AgNPs, FeNPs and Ag–FeNPs samples and (b) *H. perforatum* leaf extract.

The green-synthesized Ag–FeNPs using *H. perforatum* extract were characterized through various analytical techniques. The absorbance spectra of both the Ag–FeNP solutions and the *H. perforatum* extract were recorded using a UV-vis spectrophotometer, and the resulting spectra for all samples are presented in Fig. 3. For the synthesized AgNPs, a broad absorption band with a maximum peak at 447 nm was observed (Fig. 3a). The appearance of the characteristic surface plasmon resonance (SPR) peak for AgNPs is in good agreement with earlier studies, confirming the successful formation of silver nanoparticles.^23^ In the case of FeNPs and Ag–FeNPs, distinct absorption peaks at 356 nm and 300 nm, corresponding to metallic iron, were observed (Fig. 3a). These peaks are likely associated with Fe residues and collective surface plasmon oscillations of iron. The presence of such SPR bands in FeNPs has also been reported in FT-IR spectra in previous studies.

As illustrated, when Ag and Fe nanoparticles were analyzed individually, each exhibited its characteristic UV-vis absorption spectrum. For the bimetallic nanoparticles, while the absorption profile resembled that of FeNPs, a notable blue shift was observed along with increased peak intensity corresponding to iron as the silver content decreased. This shift is attributed to scattering effects of Fe, suggesting the formation of silver–iron core–shell bimetallic nanoparticles. Similar observations have been reported in other studies on the synthesis of bimetallic nanoparticles.^24,25^

**Fig. 4:**
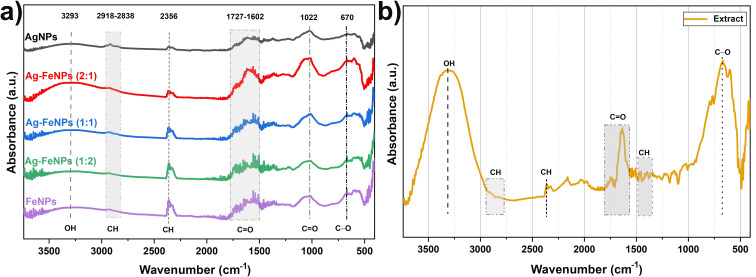
FT-IR spectra of (a) AgNPs, FeNPs and Ag–FeNPs samples and (b) *H. perforatum* leaf extract.

Fig. 4 presents the FT-IR spectra of biogenic Ag, Fe, and Ag–FeNPs synthesized using *H. perforatum* leaf extract, alongside the spectrum of the extract itself. The FT-IR profiles confirmed the presence of bioactive compounds extracted from the leaves, which are responsible for the reduction of Ag^+^ and Fe^3+^ ions and their subsequent attachment to the nanoparticle surfaces. Previous studies have reported that plant-derived flavonoids and bioflavonoid derivatives exhibit characteristic infrared signals in the O–H stretching region, aliphatic C–H vibrations, and aromatic ring modes.^26^ In the present study, the FT-IR spectra of the nanoparticles displayed absorption bands centered at 3293, 2918, 2858, 2356, 1727, 1602, 1022, 670, and 594 cm^−1^. The broad band in the 3700–3000 cm^−1^ region corresponds to O–H stretching vibrations of hydroxyl groups. Peaks observed at 2918, 2858, and 2356 cm^−1^ are attributed to aliphatic C–H stretching vibrations, while bands at 1727, 1602, and 1022 cm^−1^ are associated with C=O stretching modes. Finally, the band at 670 cm^−1^ is attributed to C–O stretching. The FT-IR spectrum of the *H. perforatum* extract closely resembled those reported in previous studies.^27,28^

Following the biosynthesis of Ag, Fe, and Ag–FeNPs, a noticeable decrease in the intensity of absorption peaks was observed compared to the original extract. This attenuation indicates the capping of the nanoparticles by the extract, the reduction of metal ions, and the stabilization of the nanoparticles during synthesis. According to several studies, these reducing and stabilizing properties are attributed to polyphenolic compounds, such as flavonoids and phenolic acids, present in the *H. perforatum* extract.^29,30^

**Fig. 5:**
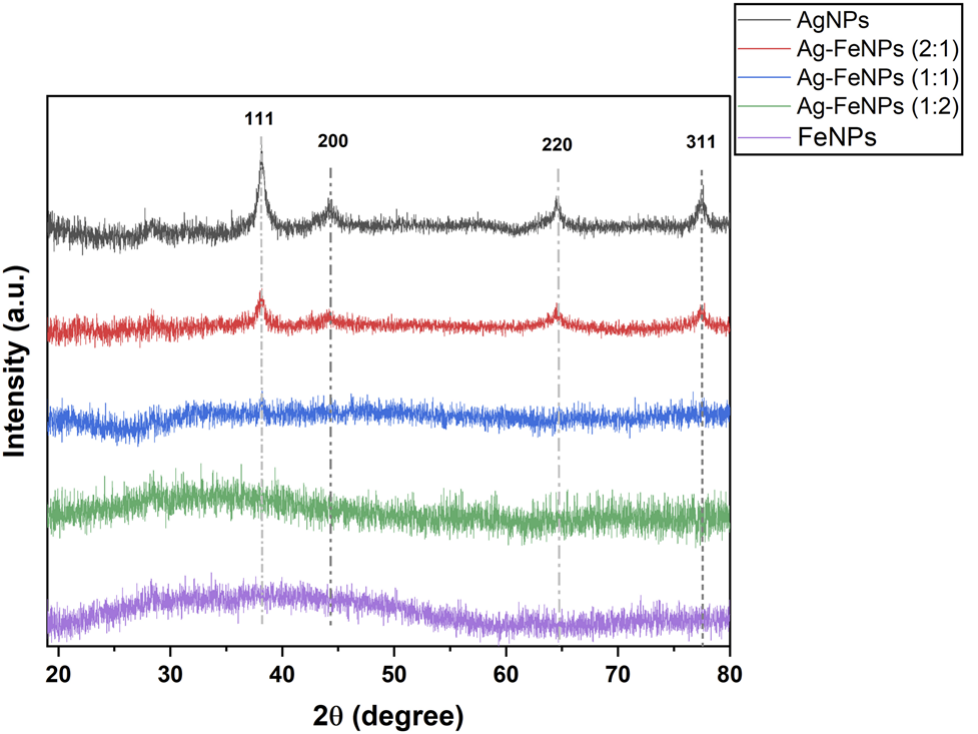
XRD patterns of AgNPs, FeNPs, and Ag–FeNPs with different Ag : Fe molar ratios. The characteristic diffraction peaks of Ag were indexed to the face-centered cubic (fcc) structure of Ag according to standard reference data (JCPDS card no. 04-0783).

X-ray diffraction (XRD) analysis was employed to determine the crystalline structures of the green-synthesized nanoparticles. The XRD patterns of Ag, Fe, and Ag–FeNPs biosynthesized using *H. perforatum* extract are presented in Fig. 5. For the AgNPs, intense diffraction peaks were observed at 2*θ* values of 38.36°, 44.34°, 64.63°, and 77.52°, corresponding to the (111), (200), (220), and (311) lattice planes, respectively of face-centered cubic (fcc) Ag (JCPDS card no. 04-0783). The average crystallite size of the Ag domains was estimated from the Ag(111) reflection using the Scherrer equation and was found to be approximately 9 nm, indicating nanoscale coherent crystalline Ag domains rather than the overall particle size.

In contrast, the XRD pattern of FeNPs did not exhibit distinct sharp diffraction peaks, suggesting a low degree of crystallinity or a highly dispersed structural state rather than a well-defined crystalline phase. This behavior is consistent with previous reports on FeNPs synthesized *via* green routes.^31,32^

In the Ag–Fe (2 : 1) nanoparticle system, a diffraction feature corresponding to the Ag(111) plane can still be discerned; however, its intensity and signal-to-noise ratio are significantly reduced compared to AgNPs. Consequently, reliable crystallite size estimation using the Scherrer equation is not feasible for this sample. From a quantitative perspective, the attenuation of the Ag-related diffraction signal indicates a pronounced suppression of effective crystallinity upon Fe incorporation into the Ag-based system.^10^

For Ag–FeNPs with higher Fe content (1 : 1 and 1 : 2 ratios), Ag-related diffraction peaks become severely suppressed or nearly indistinguishable, indicating a further reduction of crystalline order with increasing Fe concentration. No distinct diffraction peaks attributable to crystalline Fe or iron oxide phases were detected for any Ag–Fe composition, suggesting that Fe species are present in a highly dispersed and/or low-crystallinity state below the detection limit of XRD. The XRD results indicate that increasing Fe incorporation progressively suppresses the crystalline Ag phase, leading to reduced effective crystallinity rather than the absence of Ag within the bimetallic nanoparticle system.

**Fig. 6:**
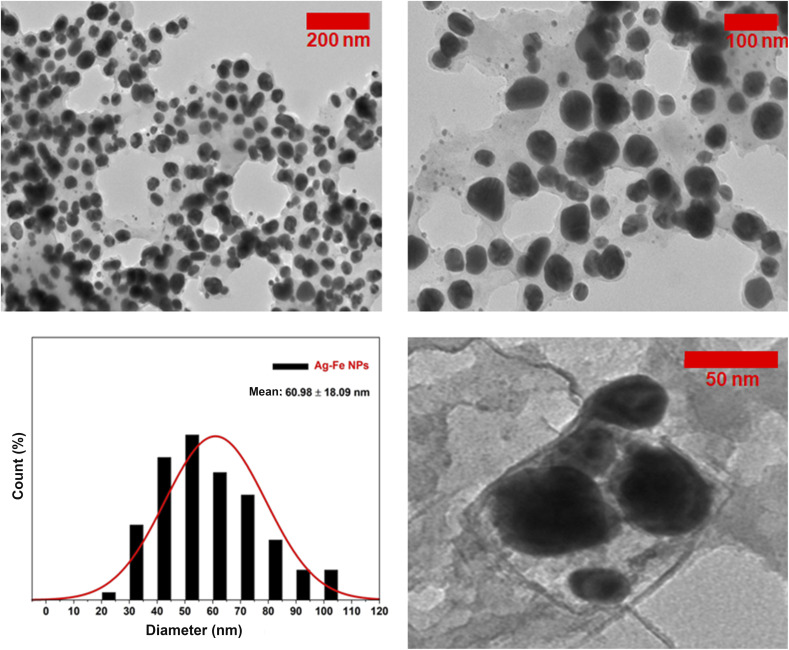
TEM images and diameter distribution histograms of Ag–FeNP (2 : 1) samples.

Transmission electron microscopy (TEM) images of the biogenic Ag–FeNPs synthesized using *H. perforatum* leaf extract are presented in Fig. 6. Based on the overall evaluation of structural (XRD) and functional performance, TEM characterization was performed exclusively for the Ag–Fe (2 : 1) nanoparticle system, which was identified as the most representative composition. For each nanoparticle sample, the diameters of 100 individual particles were measured, and the resulting size distributions were represented as histograms to indicate the average particle size (as shown in Fig. 6). The TEM analysis of AgNPs revealed spherical morphologies with a homogeneous distribution and no evidence of aggregation. The average particle size of the Ag–FeNPs was determined to be 60.98 ± 18.09 nm. These bimetallic nanoparticles were generally smaller than previously reported analogues, with particle diameters ranging from approximately 20 to 110 nm. The observed size differences are likely attributed to the use of plant leaf extract as the reducing and stabilizing agent during the green synthesis process.^33^ At higher magnifications, the TEM images of Ag–FeNPs exhibited Moiré fringes and distinct morphologies, indicative of a core–shell arrangement with a silver core and iron shell, consistent with literature reports.^34^ Moiré fringes arise due to interference between overlapping or slightly rotated crystalline layers. Such fringe patterns in the TEM micrographs confirm the crystallinity of the bimetallic Ag–FeNPs.

### 3.2. Chemical composition and morphology of GEL/CTS/Ag–Fe nanofibrous scaffold samples

**Fig. 7:**
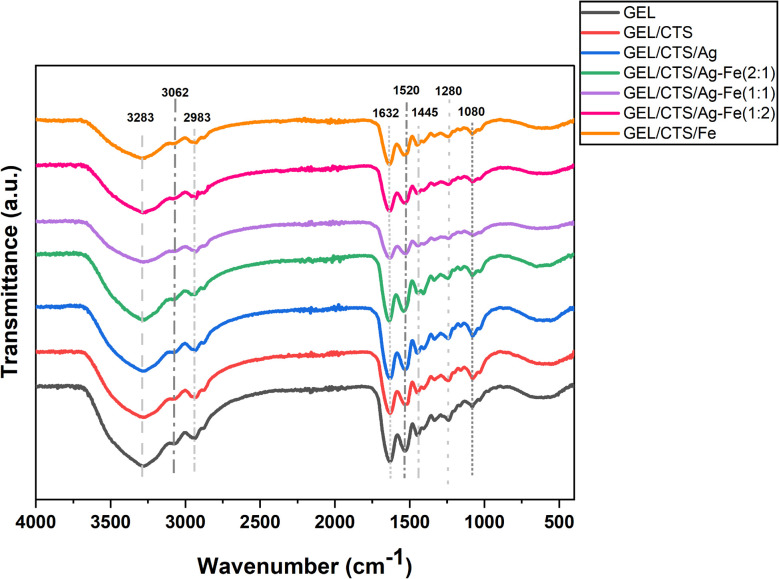
The FT-IR spectra of GEL, GEL/CTS and GEL/CTS/Ag–Fe nanofibrous scaffold samples.

Fourier-transform infrared (FT-IR) spectroscopy was performed to identify the functional groups present in the GEL, GEL/CTS, and GEL/CTS/Ag–Fe nanofibrous scaffolds, as well as to evaluate how the incorporation of each component influenced the overall polymeric matrix. The FT-IR spectra of the fabricated nanofibrous scaffolds are presented in Fig. 7. The spectra of pure GEL and GEL/CTS scaffolds exhibited several similar peaks, attributable to shared functional groups in GEL and CTS. A broad band around 3283 cm^−1^ was assigned to O–H stretching vibrations. Weak signals observed at approximately 3085 cm^−1^ and 2983 cm^−1^ corresponded to alkenyl C–H stretching and CH_2_ stretching vibrations, respectively. Strong absorption near 1632 cm^−1^ was associated with C=O stretching from amide linkages. Other characteristic bands included 1520 cm^−1^ (N–H bending coupled with C–N stretching), 1445 cm^−1^ (CH_2_ bending), 1280 cm^−1^ (N–H bending), and 1078 cm^−1^ (C–O stretching).^35^ Comparison between GEL/CTS and Ag–FeNP-loaded GEL/CTS nanofibers revealed no significant shifts in the peak positions. Only slight reductions in peak intensities and minor band shifts were observed, likely due to the low loading of nanoparticles (5 wt% relative to total polymer weight).^36^ Importantly, the incorporation of Ag–FeNPs did not induce any functional group disruption, indicating that the nanofibrous scaffolds maintained a stable chemical structure upon nanoparticle integration.

**Fig. 8:**
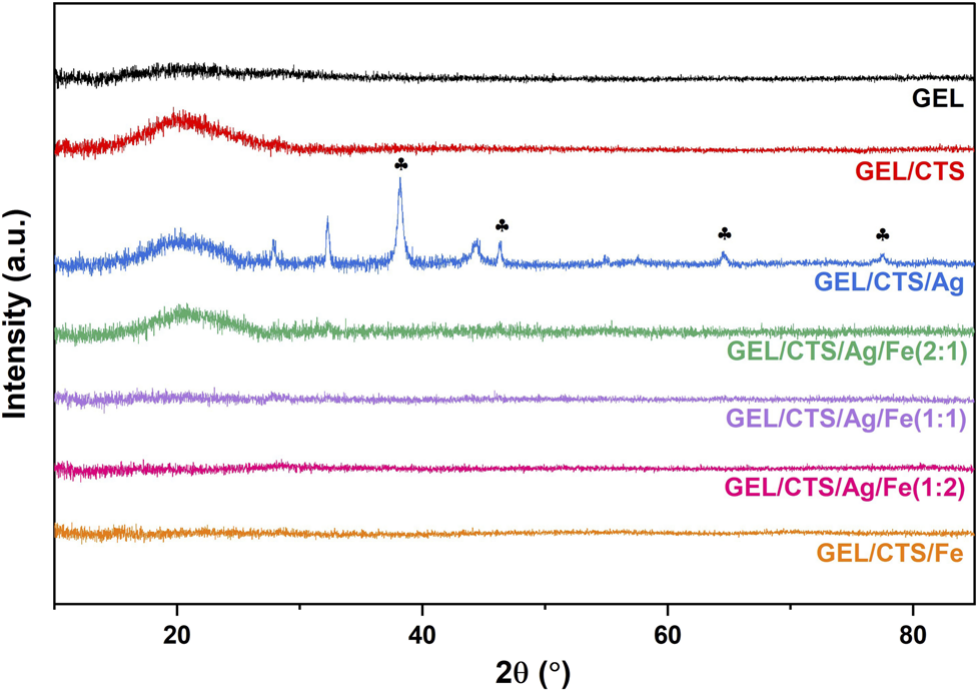
The XRD spectra of pure GEL, GEL/CTS and GEL/CTS/Ag–Fe nanofibrous scaffold samples. The weak diffraction features corresponding to Ag were indexed to face-centered cubic (fcc) Ag based on JCPDS card no. 04-0783.

X-ray diffraction (XRD) analysis was conducted to investigate the effect of Ag–FeNPs on the crystalline structure of GEL/CTS nanofibrous scaffolds. The resulting diffraction patterns are presented in Fig. 8. Pure GEL exhibited a very broad and weak diffraction profile, confirming its amorphous nature.^37^ In contrast, GEL/CTS scaffolds displayed a single broad peak between 2*θ* ≈ 15°–28°, with an increased intensity compared to pure GEL nanofibers, indicating good compatibility between the two polymer components. GEL/CTS nanofibers loaded with AgNPs showed distinct diffraction peaks at 2*θ* values of 38.23°, 44.41°, 64.63°, and 77.56°, corresponding to the (111), (200), (220), and (311) lattice planes, respectively of face-centered cubic (fcc) Ag (JCPDS card no. 04-0783). These results indicate that crystalline Ag domains remain detectable within the nanofibrous matrix despite the predominantly amorphous nature of the polymeric scaffold.

Minor low-intensity features observed at higher angles may be associated with surface-bound bio-organic species originating from the green synthesis process; however, their contribution is limited within the polymer-dominated diffraction background.

For GEL/CTS scaffolds loaded with bimetallic Ag–Fe NPs of varying compositions, an increase in Fe content resulted in a progressive attenuation of the Ag-related diffraction peaks. In particular, for the Ag–Fe (2 : 1) composition, Ag(111)-related diffraction features could still be discerned, albeit with significantly reduced intensity and signal-to-noise ratio compared to AgNP-loaded scaffolds. In nanofibrous scaffolds containing FeNPs, no distinct diffraction peaks attributable to crystalline Fe or iron oxide phases were observed, suggesting a low degree of crystallinity or a highly dispersed Fe state within the polymer matrix.

From a quantitative perspective, the weakened detectability of Ag-related diffraction features in Ag–Fe-loaded scaffolds reflects a reduction in effective crystallinity upon Fe incorporation and polymer confinement effects, rather than the absence of metallic components. Additionally, the relatively low nanoparticle loading level (5 wt% with respect to the total polymer content) inherently limits the sensitivity of XRD toward dispersed metal phases.

These results demonstrate that nanoparticle composition and polymeric confinement jointly govern the crystalline detectability within GEL/CTS nanofibrous scaffolds.

GEL, GEL/CTS, and GEL/CTS/Ag–Fe nanofibrous scaffolds were examined using field emission scanning electron microscopy (FE-SEM) to evaluate their morphology. As a natural biopolymer, GEL has been widely used in biomedical applications due to its biodegradability and biocompatibility. Previous studies have reported the fabrication of GEL-based nanofibers *via* electrospinning using polar organic solvents such as 2,2,2-trifluoroethanol and hexafluoroethanol.^38^ In tissue engineering, the electrospinnability of GEL solutions has been enhanced using various solvents and solvent mixtures, including formic acid, acetic acid, isopropanol, and water. In the present study, a fully green electrospinning strategy was employed for biomedical applications, with an acetic acid–water mixture selected as the solvent system.

**Fig. 9:**
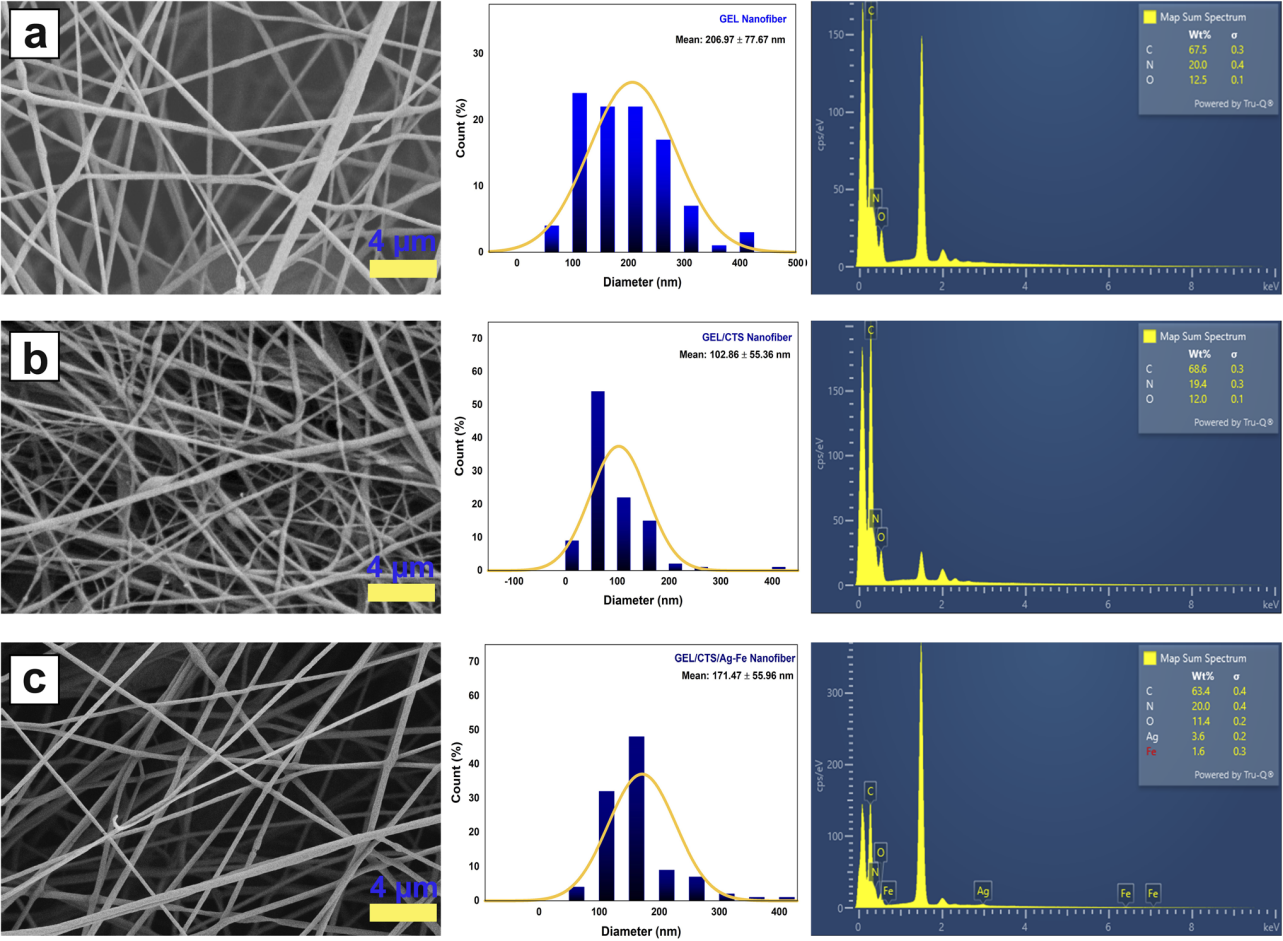
FE-SEM images, fiber diameter distribution histograms, and elemental mapping of (a) GEL, (b) GEL/CTS, and (c) GEL/CTS/Ag–FeNP (2 : 1) nanofibrous scaffolds. Elemental mapping was performed for C, O, N, Ag, and Fe.

FE-SEM images were acquired for each nanofibrous scaffold, and the diameters of 100 fibers per sample were measured using ImageJ software to determine the average fiber diameter (Table 2). The FE-SEM images, elemental mapping results, and fiber diameter histograms of CTS, GEL/CTS, and GEL/CTS/Ag–Fe scaffolds are presented in Fig. 9. Nanofiber morphology and diameters are influenced by several parameters, including solution properties and electrospinning conditions. In this study, electrospinning parameters such as applied voltage, working distance, and flow rate were kept constant for all samples. Consequently, fiber morphology was primarily determined by polymer viscosities and the type and concentration of added nanoparticles.

Pure GEL nanofibers exhibited uniform, bead-free structures with an average diameter of 206.97 ± 77.67 nm (Fig. 9a). In GEL/CTS scaffolds, the high molecular weights and viscosities of both polymers, coupled with incomplete miscibility, led to heterogeneous fiber distribution and occasional bead formation (Fig. 9b). The incorporation of CTS reduced the average fiber diameter to 102.86 ± 55.36 nm.

For GEL/CTS scaffolds loaded with Ag–FeNPs, FE-SEM analysis revealed homogeneous, bead-free fibers with an average diameter of 171.47 ± 55.96 nm. Compared to GEL/CTS fibers, Ag–FeNP incorporation increased fiber diameter and improved uniformity (Fig. 9c).

This change can be attributed to the presence and encapsulation of green-synthesized Ag–FeNPs derived from *H. perforatum* extract, which influence jet stretching behavior and polymer–nanoparticle interactions during electrospinning. When fibers loaded with Ag–FeNPs of varying Ag-to-Fe ratios were analyzed, average diameters ranged between 150 and 180 nm, decreasing with lower Ag content. This suggests that compositional variations of the incorporated nanoparticles affect the electrospinning process and resulting fiber morphology.

During electrospinning, higher charge density at the jet surface increases the electrical force, which can overcome gravitational forces and reduce the mean fiber diameter.^39,40^ This mechanism is discussed here in the context of general electrospinning behavior rather than being directly associated with specific solution conductivity changes, which were not experimentally assessed in the present study.

Elemental analysis (EDS) confirmed the presence of C, O, N, Ag, and Fe in all scaffold samples. The EDS spectra validated the incorporation of Ag and Fe on the fiber surfaces. Due to structural similarities, C, O, and N contents were comparable across GEL and CTS. In Ag–FeNP (2 : 1)-loaded fibers, the Ag content was approximately twice that of Fe, indicating uniform dispersion of bimetallic nanoparticles within the composite fibers.

### 3.3. Wettability of nanofibrous scaffolds

**Fig. 10:**
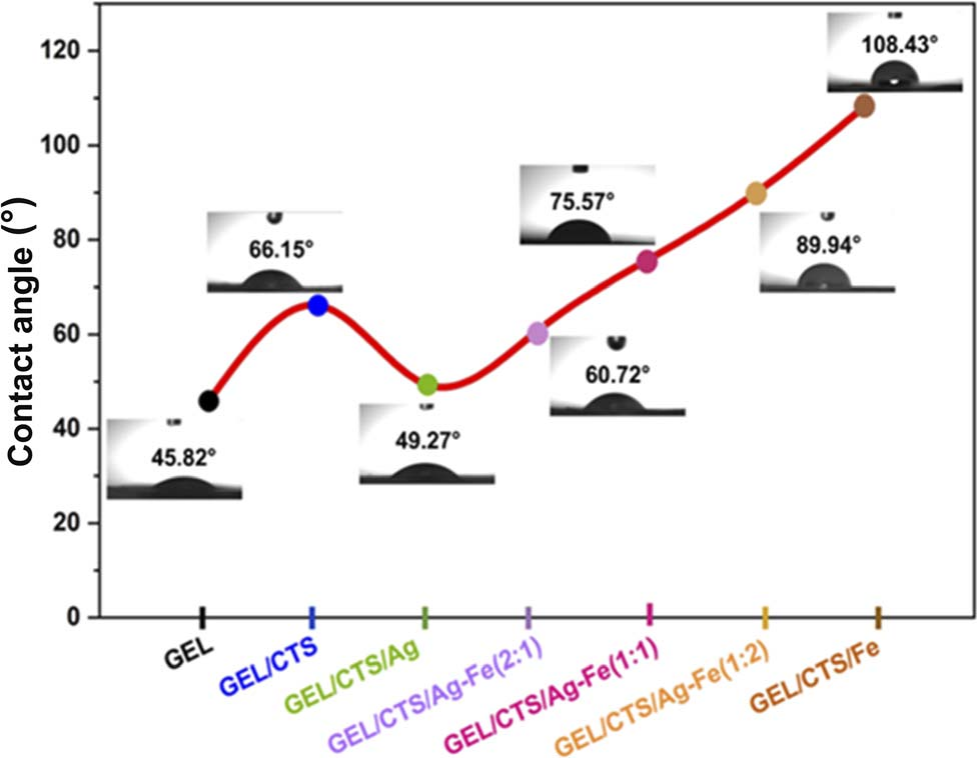
WCA measurements of GEL, GEL/CTS, and GEL/CTS scaffolds loaded with Ag–Fe NPs at varying Ag-to-Fe ratios.

The wettability of the fabricated nanofibrous scaffolds was evaluated through WCA measurements, as shown in Fig. 10. The measured contact angle values for each scaffold sample are summarized in Table 2. Scaffold hydrophilicity was assessed by analyzing the contact angles formed by water droplets on the nanofiber surfaces. According to previous reports, a WCA below 90° indicates a highly porous and hydrophilic scaffold suitable for biomedical applications, whereas a WCA above 90° corresponds to a hydrophobic surface with limited porosity and reduced applicability in tissue engineering.^41^

The GEL/CTS scaffold exhibited a WCA of 66.15°, reflecting the hydrophilic surface characteristics associated with the functional groups present in biopolymers. Among the tested scaffolds, GEL/CTS nanofibers loaded with AgNPs displayed the lowest WCA (49.27°), demonstrating that the incorporation of AgNPs enhanced the surface wettability of the scaffolds. This finding is consistent with previously reported studies.^42^

For scaffolds loaded with bimetallic Ag–FeNPs, increasing the Fe content led to a decrease in surface hydrophilicity, which can be attributed to changes in surface composition and nanoparticle–polymer interactions induced by Fe incorporation. The results indicate a transition from hydrophilic to less hydrophilic behavior depending on the Ag-to-Fe ratio, with a 2 : 1 ratio providing optimal hydrophilicity. Literature reports similarly indicate that the addition of Fe or Fe_3_O_4_ nanoparticles tends to reduce the wettability of nanofibrous mats.^43,44^

Overall, the WCA values of the Ag–Fe NP-loaded nanofibrous scaffolds ranged from approximately 49° to 89°, confirming their hydrophilic character. The hydrophilic surface of these scaffolds is particularly advantageous for cellular attachment, proliferation, and tissue integration.^45^ In the context of wound healing, hydrophilic scaffold surfaces facilitate adhesion of cells to the dressing, promoting faster wound closure and improved healing. Consequently, the enhanced wettability of the GEL/CTS/Ag–Fe nanofibrous scaffolds supports their potential applicability in various biomedical applications, including wound dressings.

### 3.4. Antibacterial test results

**Fig. 11:**
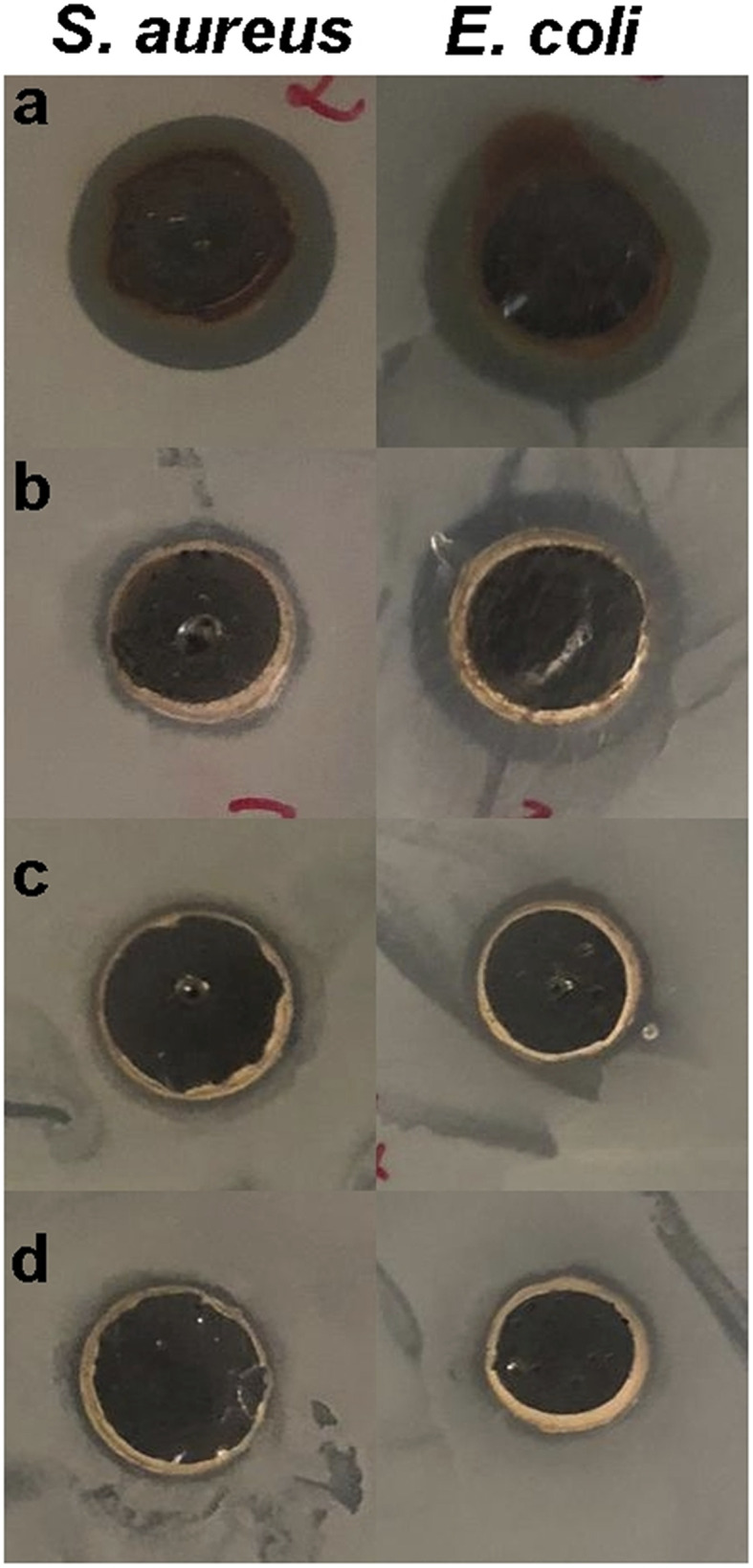
Representative agar well diffusion plates showing the antibacterial activity of synthesized nanoparticles against *S. aureus* and *E. coli*. (a) AgNPs, (b) Ag–FeNP (2 : 1), (c) Ag–FeNP (1 : 2), and (d) Ag–FeNP (1 : 1). Clear zones around the wells indicate bacterial growth inhibition.

**Fig. 12:**
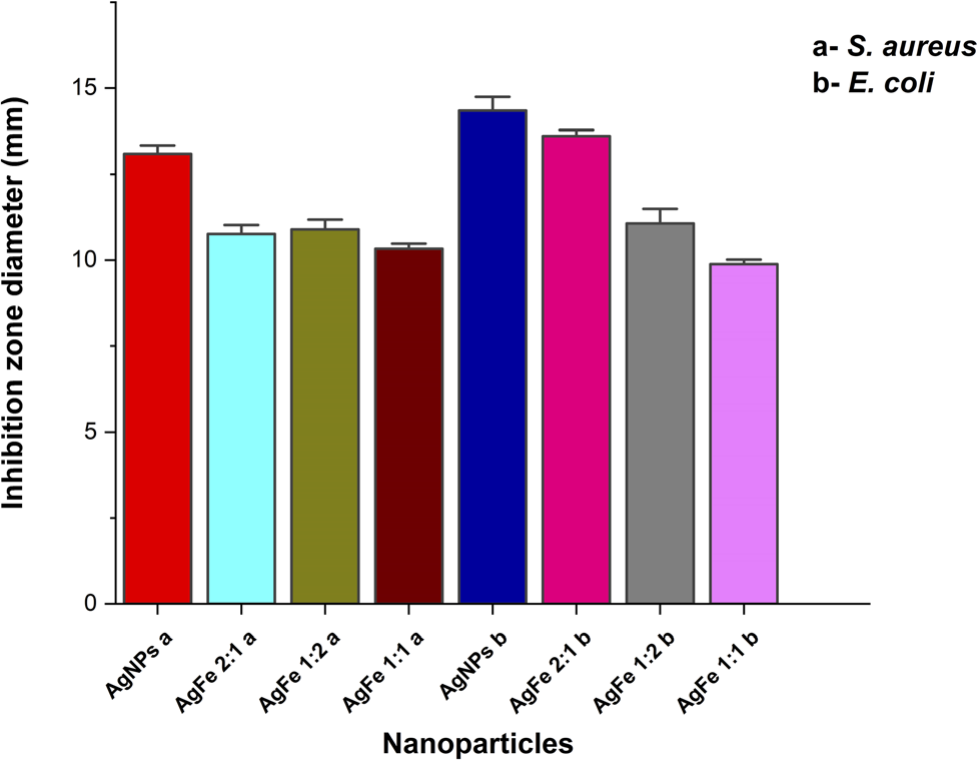
Evaluation of the antibacterial activity of synthesized nanoparticles. Inhibition zone diameters (mm) against *E. coli* and *S. aureus*. Data are presented as mean ± standard deviation (*n* = 3).

The antibacterial potential of bimetallic Ag–FeNPs with varying Ag : Fe molar ratios was evaluated against *S. aureus* and *E. coli* using the agar well diffusion assay. The efficacy was determined by measuring the diameter of the growth inhibition zones (mm), as illustrated in Fig. 11 and 12. Among the synthesized nanomaterials, pure AgNPs exhibited the most potent antibacterial activity, particularly against the Gram-negative *E. coli*, yielding a maximum inhibition zone of 14.34 ± 0.40 mm. Regarding the bimetallic compositions, the Ag–FeNP (2 : 1) ratio demonstrated the highest performance, especially against *E. coli* with a zone of 13.61 ± 0.18 mm. Conversely, the lowest antibacterial activity among the silver-containing samples was observed in the Ag–FeNP (1 : 1) composition against *E. coli*, recorded at 9.88 ± 0.14 mm. Interestingly, while the bimetallic samples showed relatively comparable results against the Gram-positive *S. aureus*, a distinct dose–response relationship was observed against *E. coli*, where the Ag–FeNP (2 : 1) significantly outperformed the Ag–FeNP (1 : 1). This suggests that a higher silver content within the bimetallic structure is critical for enhancing antibacterial activity against Gram-negative strains. Notably, pure FeNPs displayed no detectable inhibition zones against either bacterial strain, confirming that the antibacterial properties of the bimetallic system are primarily driven by the presence and synergistic integration of silver. These findings agree with Joshi *et al.* (2025), who showed that green-synthesized Ag–FeNPs are effective against bacteria.^46^ Our study adds to this by showing that the Ag–Fe ratio is very important. To keep the antibacterial effect high, a higher amount of silver is needed in the bimetallic structure to effectively stop bacterial growth.

**Fig. 13:**
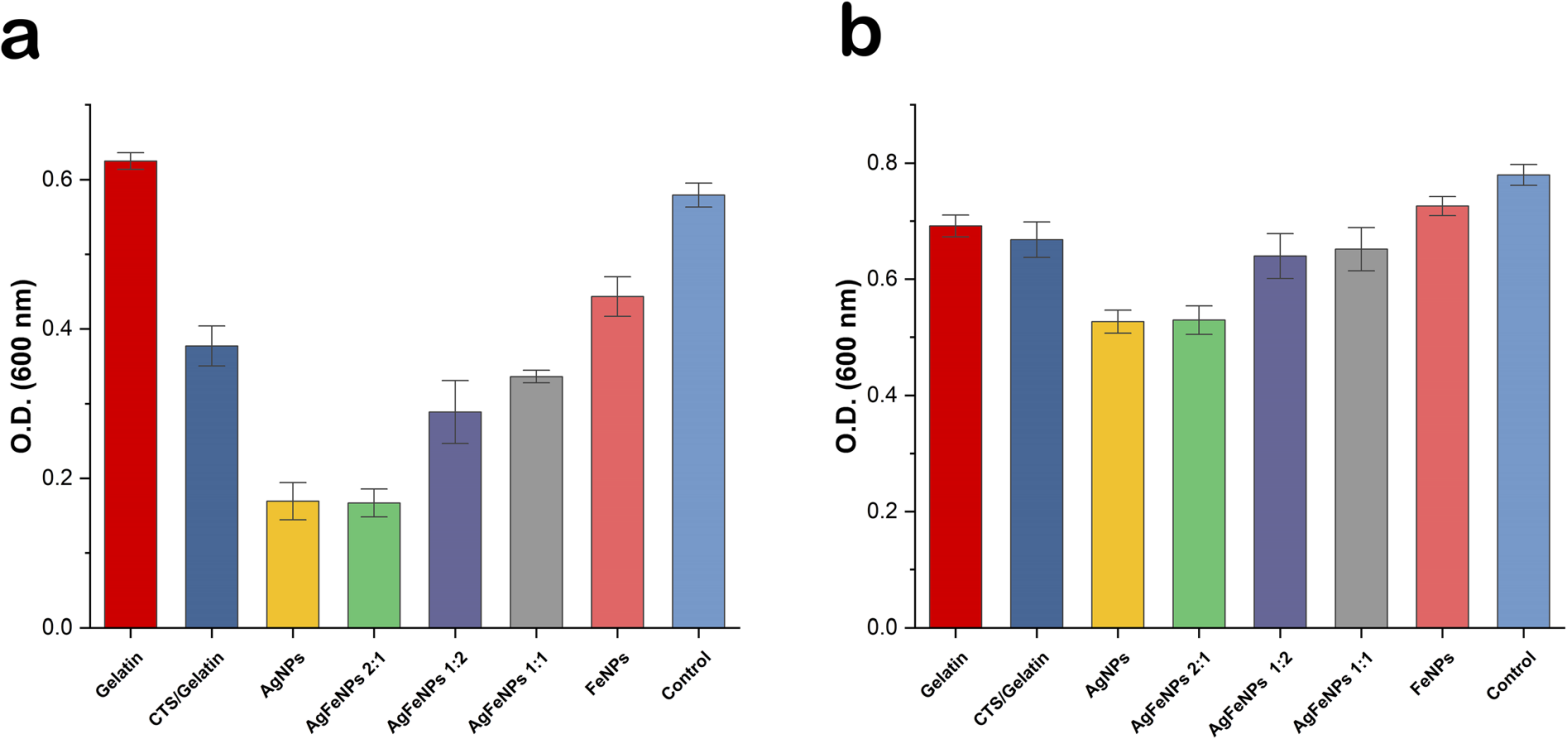
Antibacterial performance of the nanofibrous mats evaluated by optical density (O.D.) measurements at 600 nm against (a) *E. coli* and (b) *S. aureus* after 6 hours of incubation. Data are presented as the mean of triplicate measurements.

The antibacterial efficacy of the nanofibrous mats was evaluated through optical density (O.D.) measurements after a 6 h incubation period (Fig. 13). The results indicated that the nanofibers exhibited relatively limited inhibitory effects against *S. aureus*. Among the tested groups, the highest inhibition levels against this strain were observed in the AgNPs and Ag–FeNPs (2 : 1) integrated fiber samples. These findings were found to be in high correlation with the trends observed in the well diffusion assays.

The antibacterial efficacy of the synthesized nanoparticles was evaluated by determining their MIC against Gram-positive (*S. aureus*) and Gram-negative (*E. coli*) bacteria. The results, summarized in Table 3, revealed that the antibacterial potency varied significantly depending on the nanoparticle composition and the bacterial strain tested. Among all tested samples, the pure AgNPs exhibited the highest antibacterial activity, yielding the lowest MIC values of 50 µg mL^−1^ for *S. aureus* and 25 µg mL^−1^ for *E. coli*. In most cases, the nanoparticles demonstrated higher inhibitory effects against *E. coli* compared to *S. aureus*. This was particularly evident in pure AgNPs and Ag–FeNPs (2 : 1) samples. However, as the iron content increased Ag–FeNPs (1 : 1), the inhibitory effect diminished significantly, particularly against *E. coli*, where the MIC exceeded the maximum tested concentration (>200 µg mL^−1^).

**Table 3:** Minimum inhibitory concentration (MIC) values (µg mL^−1^) of AgNPs and Ag–FeNPs bimetallic nanoparticles against *S. aureus* and *E. coli*

Samples	*S. aureus*	*E. coli*
AgNPs	50	25
Ag–FeNPs (2 : 1)	100	50
Ag–FeNPs (1 : 2)	100	100
Ag–FeNPs (1 : 1)	200	>200

Also, the nanofibrous mats demonstrated significantly more pronounced inhibitory activity against *E. coli* compared to *S. aureus*. The AgNP and Ag–FeNP (2 : 1) compositions again emerged as the most effective formulations. Notably, nanofibers doped with Ag–FeNPs (1 : 1) were less active than those with Ag–FeNPs (1 : 2), reflecting a decrease in inhibitory efficacy as the proportion of silver relative to iron was altered within the matrix.

Comprehensive analysis of the results suggests that both the bare bimetallic nanoparticles and their respective nanofibrous composites are more effective against Gram-negative *E. coli* than Gram-positive *S. aureus*. The escalating global crisis of antibiotic resistance, coupled with the high economic burden of conventional antimicrobial agents, necessitates the development of efficient and cost-effective formulations.^47^

The findings of this study showed that silver remains the primary driver of antibacterial potency. However, considering the inherent toxicity of silver, synthesizing it as a bimetallic system with a more biocompatible metal like iron represents a strategic advantage. It was found that the antibacterial efficacy is not substantially compromised by this integration, and the performance in the nanofibrous mats remained almost unchanged. These observations align with existing literature reporting in silver–iron bimetallic systems.^24,48^

Collectively, these findings demonstrate a clear composition-dependent antibacterial behavior, confirming that precise tuning of the Ag : Fe molar ratio represents a critical parameter for optimizing antimicrobial performance in bimetallic nanofiber systems.

A comparison with previously reported polymer-based antibacterial wound dressing systems reveals that most nanofibrous scaffolds primarily achieve antimicrobial functionality by incorporating monometallic Ag nanoparticles into various polymeric matrices.^49^ While such approaches benefit from the well-established antibacterial efficacy of silver, they often rely on relatively high Ag contents and provide limited flexibility in compositional optimization.

In the present study, the integration of Ag–Fe bimetallic nanoparticles into a gelatin/chitosan nanofibrous matrix represents a distinct design strategy that enables compositional tuning within a biodegradable and biocompatible scaffold platform. This study showed that the Ag–Fe (2 : 1) formulation demonstrates enhanced antibacterial performance without the need for excessive silver content, while preserving favorable surface wettability and uniform fiber morphology. This efficiency points toward a synergistic reinforcement provided by the iron component. While silver is recognized for inducing intracellular oxidative stress *via* free radical pathways, its efficacy at reduced concentrations suggests that iron acts as a biochemical catalyst for membrane destabilization. Specifically, iron targets the thiol side chains of cysteine residues within the bacterial cell wall proteins. This structural compromise facilitates the penetration of Ag species even at sub-maximal dosages.^24,50,51^

The integration of a GEL/CTS polymer matrix in this study offers distinct advantages over synthetic alternatives, particularly regarding potential cytocompatibility and degradability, which are critical parameters for functional wound dressing applications.^52^ While conventional studies on Ag/PVA nanofibers emphasize that reaching high antimicrobial rates is strictly dependent on elevated silver loading densities, our GEL/CTS-based Ag–Fe bimetallic system achieves comparable efficacy through a synergistic mechanism that allows for a significant reduction in silver content.^53^ This synergy not only optimizes the antibacterial performance but also enhances the material's safety profile. In fact, similar nanofibrous systems incorporating chitosan and metal oxide nanoparticles have been reported to maintain high cell viability, suggesting that our GEL/CTS matrix—specifically engineered with a reduced silver-to-iron ratio—is likely to offer a comparable non-toxic profile for biomedical applications.^54^ Consequently, by leveraging the biochemical facilitation of iron alongside the biocompatible nature of the GEL/CTS scaffold, we provide a more sustainable and safer alternative to high-dose silver-based dressings without compromising biocidal efficiency.

## 4. Conclusions

In this study, Ag–Fe bimetallic nanoparticles were synthesized through a green approach using *H. perforatum* leaf extract and incorporated into electrospun GEL/CTS nanofibrous scaffolds for potential wound-dressing applications. The biosynthesized nanoparticles exhibited a spherical morphology with an average diameter of 60.98 ± 18.09 nm and were uniformly distributed within the polymeric network. Incorporation of CTS into GEL reduced fiber diameters, while the addition of Ag–FeNPs increased them due to nanoparticle encapsulation and reduced solution conductivity. Scaffolds containing Ag–FeNPs in a 2 : 1 Ag : Fe ratio presented favorable fiber dimensions (approximately 150–185 nm) and maintained uniform, bead-free structures.

XRD analysis confirmed the crystalline signature of AgNPs and indicated a low degree of crystallinity or a highly dispersed structural state for FeNPs, whereas increasing Fe content led to a gradual decrease in Ag-related diffraction intensities within the composite fibers. Wettability measurements showed a clear composition-dependent trend. Pure FeNP-loaded scaffolds exhibited hydrophobic behavior (WCA ≈ 108°), whereas increasing silver progressively reduced the contact angle, with Ag-containing scaffolds displaying enhanced hydrophilicity. Ag-loaded scaffolds were the most hydrophilic (WCA ≈ 49°), supporting moisture exchange essential for wound-related applications. Fe-rich compositions approached the hydrophilic threshold, while Ag-rich systems maintained lower WCA values.

Antibacterial results indicated stronger inhibition against *E. coli* than *S. aureus*, and the addition of Fe did not markedly reduce the antimicrobial efficiency of Ag-based systems. These findings suggest that Ag–FeNPs synthesized *via* a green route can provide effective antibacterial functionality while potentially reducing the need for high silver concentrations.

Overall, the Ag–Fe NPs-reinforced GEL/CTS scaffold developed here demonstrates promising characteristics as an eco-friendly, bioactive antibacterial material for potential wound-dressing applications. The results highlight that tuning the Ag : Fe ratio enables control over both surface wettability and antimicrobial performance within a sustainable fabrication framework. Future studies could focus on systematic evaluation of Ag : Fe ratios in relation to both antibacterial efficiency and cellular compatibility, including *in vitro* cell viability and proliferation assays. Additionally, integrating other bioactive agents or exploring alternative biopolymeric matrices may further enhance the functionality of these scaffolds, supporting their development toward advanced, sustainable biomedical applications.

## Author contributions

Fatma Bayram: conceptualization, supervision, methodology, characterization, data analysis, writing – original draft. Fatih Erci: conceptualization, supervision, antibacterial experiments, writing – review and editing. Nour Alhuda Asad: experimental work, synthesis, and data collection support.

## Conflicts of interest

The authors declare that they have no known competing financial interests or personal relationships that could have appeared to influence the work reported in this paper.

## Data Availability

All data supporting the findings of this study are included in the main article.
